# Supplementary material for: Inferring demographic parameters in bacterial genomic data using Bayesian and hybrid phylogenetic methods
Source: BMC Evol Biol. 2018 Jun 19;18:95. doi: 10.1186/s12862-018-1210-5 (PMC6006949; doi:10.1186/s12862-018-1210-5)
Supplement: Supplementary file 1 — Figure S1. Root-to-tip regression for all data sets. The blue points correspond to tips in the tree. The black line represents the linear regression of root-to-tip distance as a function of the sampling time. The root-to-tip distance is measured by fitting the root of the tree that maximises R2. (PDF 81 kb) [file 12862_2018_1210_MOESM1_ESM.pdf]

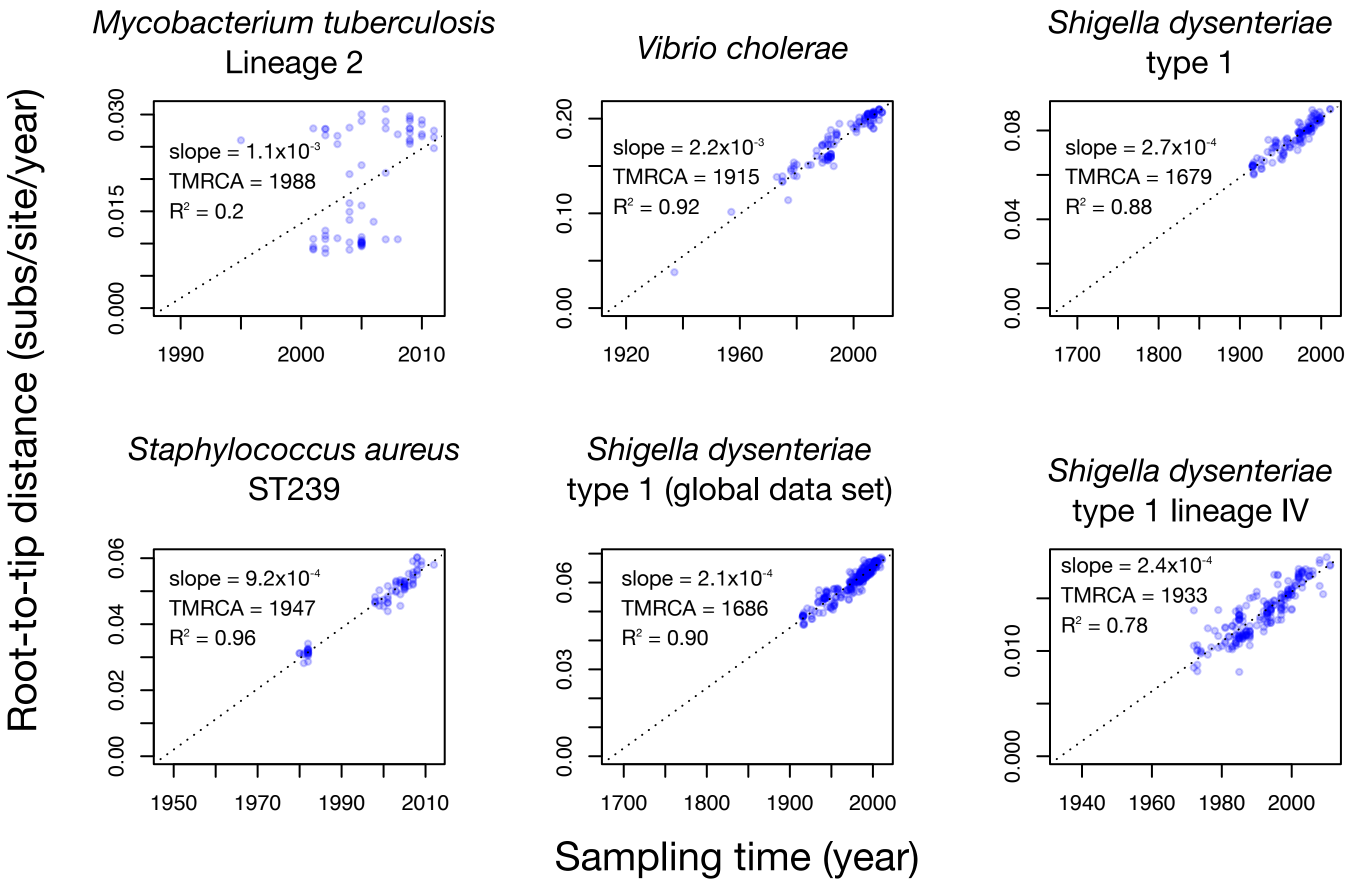

Mycobacterium tuberculosis Lineage 2
slope = 1.1x10-3
TMRCA = 1988
R2 = 0.2
Vibrio cholerae
slope = 2.2x10-3
TMRCA = 1915
R2 = 0.92
Shigella dysenteriae type 1
slope = 2.7x10-4
TMRCA = 1679
R2 = 0.88
Staphylococcus aureus ST239
slope = 9.2x10-4
TMRCA = 1947
R2 = 0.96
Shigella dysenteriae type 1 (global data set)
slope = 2.1x10-4
TMRCA = 1686
R2 = 0.90
Shigella dysenteriae type 1 lineage IV
slope = 2.4x10-4
TMRCA = 1933
R2 = 0.78
Root-to-tip distance (subs/site/year)
Sampling time (year)
